# The promise and challenge of spatial inference with the full ancestral recombination graph under Brownian motion

**DOI:** 10.1101/2024.04.10.588900

**Published:** 2025-02-17

**Authors:** Puneeth Deraje, James Kitchens, Graham Coop, Matthew M. Osmond

**Affiliations:** 1 Department of Ecology & Evolutionary Biology, University of Toronto; 2 Department of Evolution & Ecology and Center for Population Biology, University of California - Davis

**Keywords:** Ancestral recombination graph, spatial population genetics, population genetic inference, Brownian motion, networks, genetic ancestry

## Abstract

Spatial patterns of genetic relatedness among samples reflect the past movements of their ancestors. Our ability to untangle this history has the potential to improve dramatically given that we can now infer the ultimate description of genetic relatedness, the ancestral recombination graph (ARG). By extending spatial theory previously applied to trees, we generalize the common model of Brownian motion to full ARGs, thereby accounting for correlations in trees along a chromosome while efficiently computing likelihood-based estimates of dispersal rate and genetic ancestor locations, with associated uncertainties. We evaluate this model’s ability to reconstruct spatial histories using individual-based simulations and unfortunately find a clear bias in the estimates of dispersal rate and ancestor locations. We investigate the causes of this bias, pinpointing a discrepancy between the model and the true spatial process at recombination events. This highlights a key hurdle in extending the ubiquitous and analytically-tractable model of Brownian motion from trees to ARGs, which otherwise has the potential to provide an efficient method for spatial inference, with uncertainties, using all the information available in the full ARG.

## 1 Introduction

Life moves – offspring disperse, populations admix, and species’ ranges shift. While most of these events go unnoticed in the moment, they leave spatial patterns in the genetic diversity of a sample. Though often faint, we can use such signals to gain insight into the spatial history of the samples’ shared genetic ancestors.

One broad set of approaches to infer spatial history divides sample genomes up into a small number of geographic regions and, using allele frequency (e.g., Excoffier et al., 2021) or gene trees (e.g., Müller et al., 2018), estimates the split times and rates of gene flow between these regions. While useful, these approaches require the *a priori* grouping of samples and can obscure the fact that there can be population structure at many geographical scales. For example, in many species genetic differentiation builds up relatively smoothly with the geographic distance between samples. Such patterns motivate modeling approaches that treat space explicitly in so-called isolation-by-distance models. A subset of these models assume local migration between groups of individuals (demes) on a lattice (e.g., the stepping-stone model, Kimura and Weiss, 1964; Malécot, 1948), allowing the rate of increase in genetic differentiation ($F_{ST}$) with geographic distance to be used to estimate dispersal rates (Rousset, 2000, 1997). The alternative is to treat space in a truly continuous manner, avoiding the need to assume well-mixed demes or a fixed layout of individuals. At its core, the classic continuum model (Malécot, 1948; Wright, 1943) assumes lineages move as independent Brownian motions. The tractability of this model makes it useful for spatial inference, for example, inferring dispersal rate and the locations of genetic ancestors from gene trees (e.g., Lemmon and Lemmon, 2008; Novembre and Slatkin, 2009). As a result, independent Brownian motion remains a common feature of many phylogeographic methods (e.g., Dellicour et al., 2021). Further research has aimed at resolving the issues that arise when assuming lineages move as independent Brownian motions, namely the clustering of individuals in forward-in-time models (Felsenstein, 1975) and sampling inconsistency in backward-in-time models (Barton et al., 2010a). The spatial Λ-Fleming-Viot process (Barton et al., 2010a, 2013, 2010b) is an alternative that, among other things, incorporates local density dependence to avoid these issues. However, the model’s mathematical complexity makes inference computationally expensive (Wirtz and Guindon, 2023) and therefore limited to small sample sizes. Thus, independent Brownian motion, despite its limitations, continues to be an analytically tractable and computationally feasible model that is often useful for spatial inference, at least when dealing with non-recombining sequences.

On the empirical front, the increasing feasibility of whole-genome sequencing has led to an influx of genetic data and motivated advances in the inference of the genealogical history of a sample undergoing recombination (e.g., Deng et al., 2024; Kelleher et al., 2019; Rasmussen et al., 2014; Schaefer et al., 2021; Speidel et al., 2019; Wohns et al., 2022). Recombination allows different regions of the same chromosome to have different gene trees. Although single-tree approaches may be suitable when studying non-recombining sequences (e.g., mitochondrial DNA), they do not capture the range of genetic relationships found across recombining genomes nor the correlations between these relationships. For this, we must turn to the ancestral recombination graph (ARG).

An ARG encodes the complete genetic history of a sample of recombining genomes (Griffiths and Marjoram, 1996; Hudson, 1983; Lewanski et al., 2023). As such, it has proven to be an incredibly rich source of information about the history of the sample (Harris, 2019; Hejase et al., 2020; Lewanski et al., 2023). One particularly promising application is spatial inference. Two recent methods use an ARG to provide point estimates of ancestor locations (Grundler et al., 2024; Wohns et al., 2022) while another provides the full probability distribution for an ancestor’s location but ignores correlations between trees (Osmond and Coop, 2024). Here, we extend the classic Brownian motion model for trees to describe movement down an ARG. Under this model, we derive the full likelihood of the sample locations given an ARG, allowing us to infer the dispersal rate and the probability distribution for the location of every genetic ancestor in the ARG using the complete genealogical history of the sample. In doing so, we highlight a key problem in extending a classic model of movement from trees to ARGs.

## 2 Brownian motion on an ARG

An ARG is a graphical representation of the genealogical history of a set of sample genomes that may have undergone recombination in the past (Wong et al., 2023). As the history of the samples at each site in the genome can be depicted as a tree, an ARG weaves together these histories based upon their shared structure. Altogether, an ARG provides the complete genetic history of the samples across the genome.

Each node in an ARG represents a haploid genome. Edges are directed from an ancestral node to a descendant node, describing the line of inheritance over time. A node that is the product of recombination has two ancestral nodes with annotated edges referring to the specific regions of the genome that were inherited from each. Time is measured backwards from the present, starting from the most recent sample node at time t=0 and increasing as we go deeper into the past towards the root of the ARG, its grand most recent common ancestor (GMRCA). In this work we focus on “full ARGs” (Wong et al., 2023), which include the complete set of coalescent, recombination, and common ancestor nodes. This is mainly for ease of explanation. Our conclusions and algorithms are applicable more broadly to commonly inferred “simplified ARGs”, which only include coalescent nodes found in the local trees (Baumdicker et al., 2022; Lewanski et al., 2023; Shipilina et al., 2023; Wong et al., 2023).

We model the movement of genetic material forward in time by Brownian motion with dispersal rate $\sigma^{2}$ (see Table 1 for a list of key symbols). In other words, we assume that the location of a node is normally distributed about its parent node with variance $\sigma^{2}t$, where t is the length of the edge connecting them (in generations). In each generation, autosomal inheritance is equally likely via the mother or father; therefore, the effective variance is the average of maternal and paternal variances (e.g., Smith et al., 2023). While the computations are shown for one dimension, they are readily extended to two dimensions by replacing the dispersal rate, $\sigma^{2}$, with a dispersal matrix, $\Sigma =\left[\begin{array}{cc} \sigma_{x}^{2} & \sigma_{xy}\\ \sigma_{xy} & \sigma_{y}^{2} \end{array}\right]$, where $\sigma_{xy}$ is the covariance in offspring locations across the two dimensions.

We assume we know the ARG with certainty and therefore all edges are treated equally regardless of the amount of genome they span; if we know with certainty that an edge exists in the graph, we do not care how much genetic material those ancestors contributed to the samples. This differs from the approach of Grundler et al. (2024), which gives less weight to edges with smaller spans due to the lower confidence in accurately inferring these short span edges from genetic data. Here we are more focused on developing the underlying theory rather than pragmatically applying a method.

All of the equations presented below are generalizations of the standard tree-based approach to account for recombination. As a tree is simply an ARG without recombination, these generalized equations will all collapse back to those previously presented in literature for a single tree.

The first step in estimating dispersal rates and the locations of genetic ancestors given an ARG is calculating a likelihood for the sample locations. Under Brownian motion, the vector of sample locations, $\vec{L}$, has a multivariate normal distribution, specifically,

(1) $$\vec{L}\sim 𝒩\left(\mu 1_{n_{s}},\sigma^{2}S\right),$$

where μ is the location of the root (GMRCA) of the ARG, $1_{n_{s}}$ is a column vector of length $n_{s}$ (number of samples) consisting of just ones, $\sigma^{2}$ is effective the dispersal rate, and $\sigma^{2}S$ is the covariance matrix between the sample locations. We refer to S as the sample matrix and it represents the covariance between the samples that is solely due to the structure of the ARG, independent of the dispersal rate.

For a single tree we can assume that the displacement along any edge is independent of the other edges’ displacements. The displacement of an edge from node i to node j is distributed as $D_{i,j}\sim 𝒩\left(0,\sigma^{2}t_{i,j}\right)$, where $t_{i,j}$ is the length of the edge in generations. A sample’s location is given by the sum of the displacements along the edges from the root to the sample. We refer to a series of edges connecting an older (further in the past) node to a more recent node as a path. We are particularly interested in paths from the root to a sample and call the set of such paths the sample paths. The covariance between the locations of two samples is then simply $\sigma^{2}$ times the shared time between the unique corresponding sample paths, hence the entries of S are just the shared time between each pair of samples, as mentioned above.

**Figure 1:**
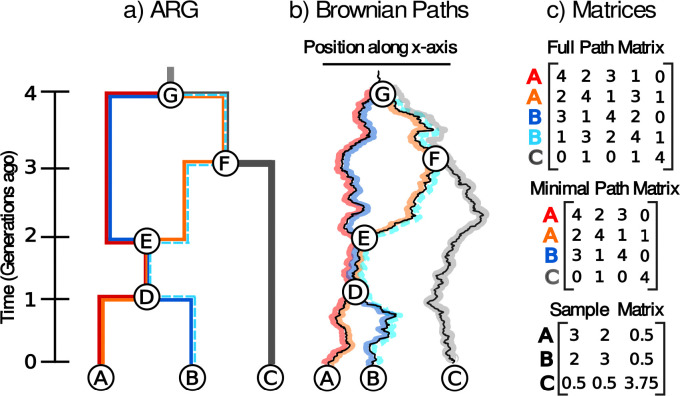
Brownian motion on an ARG. (a) An ARG, (b) an instance of Brownian motion along the ARG, and (c) the corresponding path ($S_{p}$) and sample (S) matrices. Sample paths through the ARG are individually colored. Samples A and B are associated with two paths each - red/orange and navy/sky blue, respectively - because they are beneath a recombination node (node E). In the Brownian motion model illustration, note that the paths meet precisely at the recombination node E. The path matrices contain the shared time between the sample paths. The sky blue path from sample B is excluded when calculating the minimal path matrix as it provides redundant information.

In generalizing this approach to an ARG we run into two related issues. First, each recombination node creates a loop within the graph. This means that samples found below a recombination node will have multiple paths back to the root. Therefore, computing the entries of S for an ARG is not as straightforward as it is for a tree and the covariance between samples must take into account this braided history where multiple paths lead to the same sample. Second, the presence of these loops means that we can no longer always assume that the displacements along edges of the graph forward in time are independent. Assuming that the parents of a recombination node must precisely meet each other in space, the displacement along the edges involved in the loop must satisfy the condition that the sum of the displacements around the left half of the loop equal the sum around the right half. For example, in Figure 1 the displacement from node G to node F plus the displacement from node F to node E must equal the displacement from node G directly to node $E,D_{G,F}+D_{F,E}=D_{G,E}$. We refer to the collection of these conditions across an ARG as $\eta_{\text{loops}}$.

To account for these conditions, $\eta_{\text{loops}}$, we start by assuming (incorrectly) that Brownian motion along each edge of the ARG is indeed independent, as in the case of trees. This leads to a sample node having different location distributions based on the choice of sample path. Nevertheless, we encode the distribution of locations of the nodes at the tips of each sample path in $\vec{L_{p}}$, a column vector of length $n_{p}$ (number of sample paths) with distribution

(2) $$\vec{L_{p}}\sim 𝒩\left(\mu 1_{n_{p}},\sigma^{2}S_{p}\right),$$

where $S_{p}$, referred to as the path matrix, is the matrix of shared times between sample paths. To get the correct distribution of sample locations, $\vec{L}$ (Equation 1), we need to condition $\vec{L_{p}}$ so that all sample paths that end at the same sample also end at the same location. We refer to this condition as $\eta_{\text{paths}}$ and show that $\eta_{\text{paths}}$ reduces to $\eta_{\text{loops}}$ (Section S2). The sample matrix can then be easily computed from the path matrix (Section S1),

(3) $$S={\left(P^{T}S_{p}^{g-}P\right)}^{-1}.$$

Here, $S_{p}^{g-}$ is the generalized inverse of $S_{p}$ and P is a conversion matrix of size $n_{p}\times n_{s}$ that pairs each sample path i with its associated sample j. The i,j^*th*^ element of P is 1 if path j starts at sample i, otherwise it is 0. As a check, note that the sample matrix and path matrix are equivalent for a tree as each sample is associated with only one path.

We can now compute the likelihood of sample locations (Equation 1). Though this conversion from $S_{p}$ to S is possible, it is generally unnecessary and it is anyways easier to work with the path matrix in what follows. Additionally, while we could calculate the shared times between every pair of sample paths through the ARG, many of the paths are not linearly independent from one another and are therefore redundant in our calculations. In practice, we use only a minimal subset of paths and its corresponding minimal path matrix (see Figure 1 for an example). While the total number of sample paths grows faster than linearly with the number of recombination events, the minimal number of sample paths grows linearly (being equal to the number of samples plus the number of recombination events; see Section S3). We have developed an algorithm to calculate the minimal path matrix in a single tip-to-root traversal of the ARG (Section S3).

### 2.1 Multiple roots

For multiple reasons we might want to focus on just the recent past. First, long-term movement may not be accurately captured by Brownian motion due to geographic boundaries or large-scale population movements (Ianni-Ravn et al., 2023). Second, as we move further back into the past, sample lineages can become spatially well mixed (Wakeley, 1999), which makes it difficult or impossible to extract meaningful spatial information about the deep history of samples. Third, with ARG inference, deeper nodes in the ARG are often poorly resolved, both in timing and topology (e.g., see Figure S2B in Fan et al., 2023). To avoid these issues, we will often want to cut off an ARG at a given time in the past and ignore any deeper connections.

When we chop an ARG below the GMRCA the graph no longer has a single root (Figure S1). Instead there are multiple roots, i.e., nodes with no parent nodes, each associated with specific paths through the ARG. Let $\vec{\mu }$ be the vector of root locations (of length $n_{r}$, the number of roots) and R be a conversion matrix of size $n_{p}\times n_{r}$. The i,j^*th*^ element of **R** is 1 if path j terminates at root i, otherwise it is 0. Then $R\vec{\mu }$ is the vector of root locations associated with each of the $n_{p}$ paths.

The full covariance between the locations of the samples at the ends of the sample paths is the covariance created by the structure of the ARG plus the covariance between the root locations, $\sigma^{2}(S_{p}+Cov(R\vec{\mu },R\vec{\mu }))$. Here, we assume that the root locations are independent of each other and that each has zero variance, $Cov(R\vec{\mu },R\vec{\mu })=0$, so that the covariance between the locations of the samples at the ends of the sample paths remains $\sigma^{2}S_{p}$. The assumption of independence is reasonable if we cut off the ARG at a point by which the ancestors are well mixed in a finite habitat. The assumption of zero variance in root locations can be relaxed by adding a variance to the covariance term between pairs of paths starting at the same root. The resulting likelihood of sample locations is

(4) $$\vec{L_{p}}\sim 𝒩(R\vec{\mu },\sigma^{2}S_{p}).$$

### 2.2 Estimates

If $R^{T}S_{p}^{g-}R$ is invertible, we can compute the MLEs for the root locations given the observed sample locations, $\vec{\ell^{*}}$, as the unique solution to (Section S1.2)

(5) $$\hat{\vec{\mu }}={\left(R^{T}S_{p}^{g-}R\right)}^{-1}RS_{p}^{g-}P\vec{\ell^{*}}.$$

Once we have estimated the locations of the roots, we can compute the MLE of the dispersal rate,

(6) $$\hat{\sigma^{2}}=\frac{{\left(P\vec{\ell^{*}}\;-\;R\hat{\vec{\mu }}\right)}^{T}S_{p}^{g-}(P\vec{\ell^{*}}\;-\;R\hat{\vec{\mu }})}{n_{s}}.$$

With estimates of both the root locations and the dispersal rate, we can then calculate the distribution of any internal node location (a genetic ancestor). Given an internal node, we choose an arbitrary path from that node to one of the roots. Conditional on the dispersal rate, root locations, and observed sample locations, the location of an internal node, $L_{a}$, is normally distributed with mean

(7) $$E[L_{a}|\hat{\vec{\mu }},\hat{\sigma^{2}}]={\hat{\mu }}_{r_{a}}+{\vec{s_{a}}}^{T}S_{p}^{g-}(P\vec{\ell^{*}}-R\hat{\vec{\mu }}),$$

where $\vec{s_{a}}$ is the vector of shared times with the minimal paths used to construct $S_{p}$ and ${\hat{\mu }}_{r_{a}}$ is the MLE location of the ${r_{a}}^{th}$ root (the ${r_{a}}^{th}$ element of $\vec{\mu }$). The total variance in the internal node’s location is a combination of the variance due to Brownian motion and the variance due to uncertainty in the root locations,

(8) $$Var\left(L_{a}\right)=\hat{\sigma^{2}}V,$$

where

(9) $$V=\underset{\text{due}\;\text{to}\;\text{Brownian}\;\text{motion}}{\underbrace{t_{a}-{\vec{s_{a}}}^{T}S_{p}^{g-}\vec{s_{a}}}}+\underset{\text{due}\;\text{to}\;\text{uncertainty}\;\text{in}\;\text{root}\;\text{locations}}{\underbrace{{\left({\vec{e}}_{r_{a}}-R^{T}S_{p}^{g-}\vec{s_{a}}\right)}^{T}{\left(R^{T}S_{p}^{g-}R\right)}^{-1}({\vec{e}}_{r_{a}}-R^{T}S_{p}^{g-}\vec{s_{a}})}},$$

and ${\vec{e}}_{r_{a}}$ is a length $n_{r}$ column vector that is zero everywhere except with a 1 at the ${r_{a}}^{\text{th}}$ position.

## 3 Building intuition

### 3.1 Ancestor locations

**Figure 2:**
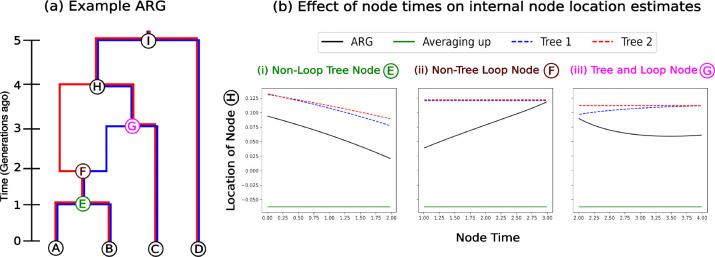
Ancestor locations. (a) Toy 2-tree ARG. (b) Most likely location of node H computed using the ARG (black), individual trees (red and blue), and the averaging-up method (green), plotted as a function of the time of (i) a node that alters individual trees and the ARG but is not part of the ARG loop, (ii) a node that doesn’t alter individual trees but is part of the ARG loop and (iii) a node which is part of the ARG loop that alters a tree and the ARG. Locations of nodes A,B,C and D are −0.5, 0, 0.5 and 1, respectively.

Changing either the topology or branch lengths within an ARG will in general change the dispersal rate and ancestral location estimates. We show the latter in Figure 2, where we compare against two previous approaches: 1) the MLE location with Brownian motion on each marginal tree (as in Osmond and Coop, 2024) and 2) simple averaging, where a node’s location is the average of its child nodes’ locations (as in Wohns et al., 2022, except we use the full ARG instead of the simplified one). By separately altering the timing of various nodes in the graph (E,F, and G), we observe a change in the MLE location of node H under our model. In contrast, point estimates from the averaging method are unaffected by changes to node timing (green lines in Figure 2b), as this approach does not incorporate edge lengths. Meanwhile, tree-based MLEs (red and blue curves in Figure 2b) respond to changes in the timing of a node only if it affects the edge lengths in the given tree (e.g., the timing of node G only affects shared times in the blue tree). Recombination nodes (e.g., node F) do not affect the shared times in trees and so their timing does not affect tree-based estimates. When the timing of a node affects a tree and is in a loop (e.g., node G), the ARG and tree estimates can show opposite trends (black vs. blue curves in Figure 2b–iii). Note that under a model of Brownian motion, moving node G further into the past should move the location of node H towards the locations of nodes A and B and away from the location of node C (as it allows more time for the node C to wander away). This behavior is only captured when using Brownian motion on the ARG, not just on an individual tree.

**Figure 3:**
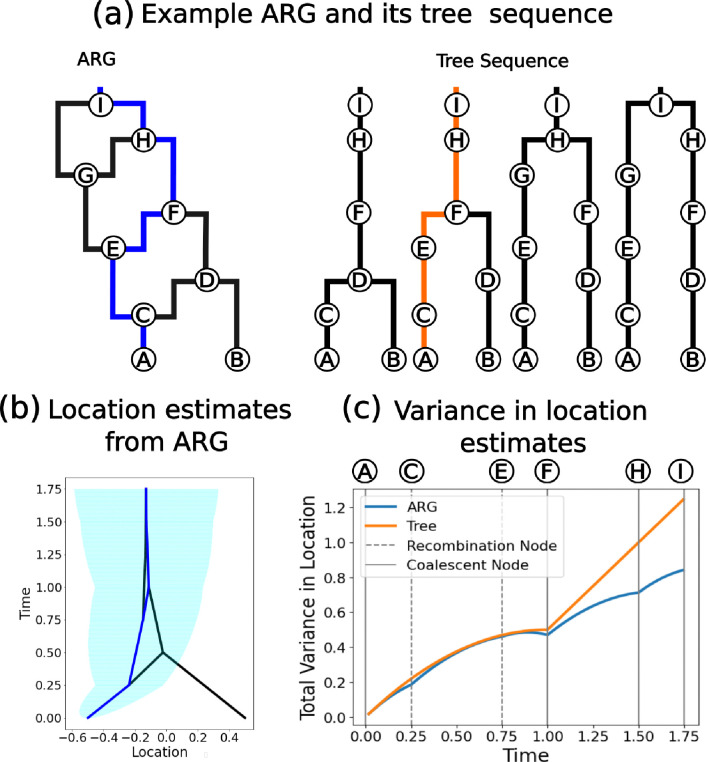
Uncertainty in ancestor locations. (a) Example ARG and the equivalent tree sequence representation with the same path highlighted. (b) The most likely location estimates (lines) and the 95% confidence interval of the blue path (shading) using the ARG. (c) The variance in location estimates, assuming $\sigma^{2}=1$, along the highlighted path using the full ARG (blue) and using just the single tree (orange).

With the greater amount of information contained within the ARG vs. a single tree, we always estimate ancestor locations with higher certainty than the tree-based method provided we use the same dispersal rate (Figure 3c). In the absence of any other information, the variance in the location estimate increases linearly with time under Brownian motion. Both coalescent and recombination nodes bring additional information and hence reduce the increase in uncertainty of the location estimates as the lineage is tracked backwards in time (Figure 3c). Larger ARGs (either due to more samples or more trees) would have more such nodes bringing in extra information and so would show a greater reduction in variance compared to a single tree.

### 3.2 Dispersal rate

**Figure 4:**
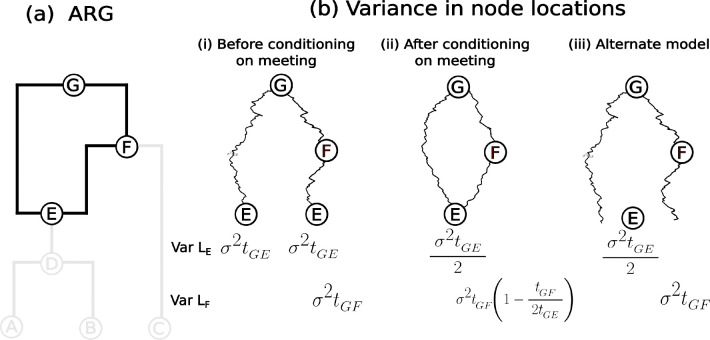
Variance in sample locations. (a) The ARG with the loop highlighted. (b) The variances of nodes E and F of the loop (i) before conditioning on the two paths along the loop meeting at E, (ii) after conditioning on them meeting and (iii) for an alternate model, where E is located at the average of the locations of its two parents.

**Figure 5:**
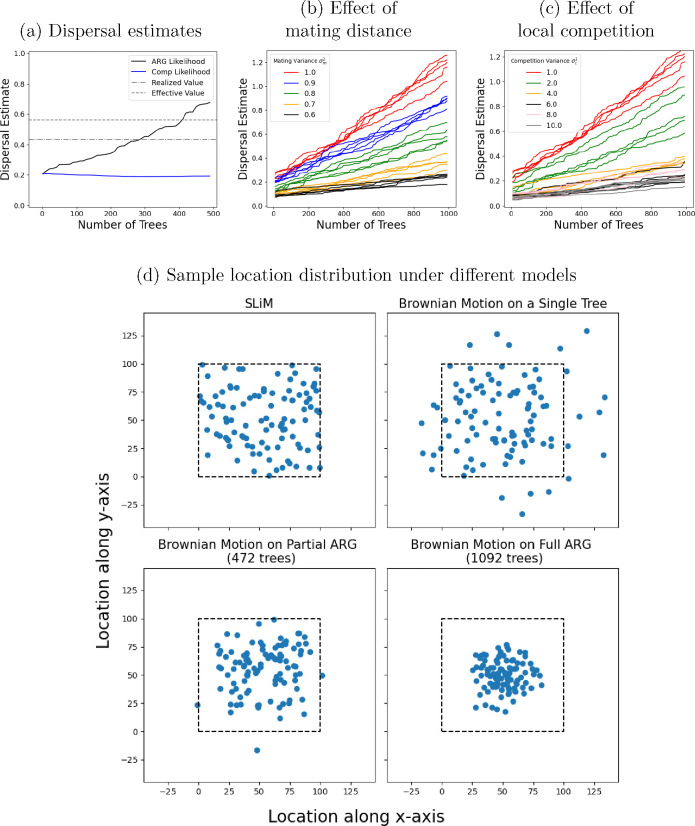
Dispersal rate accuracy. (a) Dispersal rate estimates as a function of the number of trees in the ARG used in the computation. We compare the maximum composite likelihood estimate over marginal trees (blue) and the maximum likelihood estimate from the ARG composed of the same set of trees (black). The dashed line is the effective dispersal rate (averaged over maternal and paternal variances) used in the simulation. The dashed-dotted line is the realized dispersal rate in the simulation (average squared displacement per time over all edges), which accounts for habitat boundaries. (b-c) ARG maximum likelihood dispersal estimates as a function of the number of trees colored by the variance in the (b) mating kernel and (c) competition kernel (5 replicates each).(d) The expected distribution of sample node locations under four different models. In the top left we show the sample locations from a replicate of the individual-based simulation. We then use this simulated ARG to draw sample locations from a model of Brownian motion down a single tree (top right), down a portion of the ARG (bottom left), and down the full ARG (bottom right). The dotted line is the habitat boundary of the simulations.

For a given number of samples, each additional recombination event in the ARG leads to a more clustered distribution of sample locations in the forward-in-time model. This means that, for a given set of sample locations, each additional recombination event in the ARG increases the dispersal estimate. We demonstrate the increased clustering of sample locations under the forward-in-time model in Figure 4. Focus on the variance in the location of the recombination node, E, and first consider the two paths to E(G→E and G→F→E) independently. The variance of the location of E along either path is $\sigma^{2}t$, where t is the time between G and E, which corresponds to the variances in the location of E in each of the two the marginal trees. However, upon conditioning on the loop, $\eta_{loops}$, the variance in the location of E reduces to half of its unconditioned variance, $\sigma^{2}t/2$. More generally, we can treat the loop (G→E→F→G) as a Brownian bridge from G back to G and therefore calculate the variance at any point along the loop X as $\frac{t_{X,G}\left(2t-t_{X,G}\right)}{2t_{X,G}}$ where $t_{X,G}$ is the time between X and G (for instance, $t_{E,G}=t$) (Chow, 2009, Eq 12). Intuitively, two lineages starting at the same point are more likely to meet again if they do not wander too far away from one another. This cascades down to reduce the variance of the sample locations below recombination nodes, leading to more clustered sample locations (see Section S5 for a proof and Figure 5c for simulations).

This clustering also occurs, though to a lesser extent, in an alternate model of Brownian motion on an ARG (Bastide et al., 2018), which allows the parents of E to be any distance apart and places E at their midpoint (Figure 4b–iii). Here, for a single loop, the variance of the location of the recombination node is the variance in the average of its parents locations, $Var\left(\left(\sigma^{2}t_{G,E}+\sigma^{2}t_{G,E}\right)/2\right)=\sigma^{2}t_{G,E}/2$, just as in our original model. The difference is that, unlike in the original model, the variance along the parental lineages (e.g., node F), and consequently the variance along lineages branching off from the loop (node C), are not reduced.

## 4 Testing the theory

### 4.1 Simulations

To assess the accuracy of estimates from this approach we perform individual-based two-dimensional spatial simulations using SLiM v4.0 (Haller and Messer, 2023), extending those run by Osmond and Coop (2024). Additional one-dimensional simulations and analyses are described in the supplementary materials (Section S8). Simulations include density-dependent reproduction and a finite habitat boundary. Neither of these characteristics is captured directly under the assumptions of Brownian motion, though Osmond and Coop (2024) show that despite these differences, dispersal rates and ancestral locations can be accurately estimated under this model when applied to individual trees.

Simulations start with 10,000 individuals uniformly randomly distributed in a 100 × 100 unit area, with each individual being diploid for a 1 megabase chromosome. All individuals are hermaphrodites. In each generation an individual acts once as a mother, choosing its mate randomly based on distance (we assume a Gaussian mating kernel with variance $\sigma_{m}^{2}$ within a radius of $3\sigma_{m}^{2}$. The number of offspring for each mating pair is a Poisson random variable with mean $\lambda =\frac{2}{1+C}$, where C is the sum of the interaction strengths, also Gaussian with variance $\sigma_{c}^{2}$, with neighbors within a radius of $3\sigma_{c}^{2}$. When there are no mates within the interaction distance no offspring are produced. Offspring are placed relative to their mother’s position with a normal random variable offset in each dimension with variance $\sigma_{d}^{2}$. If the offset would place the offspring outside of the area, the offset is reflected off of the boundary wall and back into the area. Note that the effective dispersal rate, the expected variance in the distance between the offspring and either parent not accounting for the reflections, is given by $\sigma_{d}^{2}+\frac{1}{2}\sigma_{m}^{2}$ (Smith et al., 2023); the realized dispersal rate, accounting for the reflecting boundaries, will be lower. The locations and relationships between individuals are recorded in a <monospace>tskit</monospace> tree sequence, which is saved at the end of the simulation (Haller et al., 2019). Unless otherwise stated, the tree sequences are simplified to remove nodes that do not affect the broader topology of the graph and the ARG is chopped at 2000 generations in the past.

### 4.2 Dispersal rate

We first estimate dispersal rates from the simulated ARGs using Equation 6 and compare these estimates to the effective and realized dispersal rates from the simulations. We also compute the average dispersal rates calculated over the individual trees, which is identical to the composite likelihood method of Osmond and Coop (2024) without importance sampling. Consistent with previous work (e.g., Ianni-Ravn et al., 2023; Kalkauskas et al., 2021), the dispersal estimate from the composite likelihood over trees underestimates the simulated values (blue curve in Figure 5a) due to habitat boundaries. The average dispersal estimate over all trees stabilizes as we incorporate more trees (i.e., more of the chromosome). In contrast, the dispersal estimate from our ARG likelihood systematically increases as we include more trees, starting as an underestimate but eventually leading to an overestimate of the true dispersal rate (black curve in Figure 5a).

To understand the cause of this increase in the dispersal rate estimates under our model, recall that the displacements along edges in a loop are constrained as both sides of the loop need to come back together again at a recombination node. As we saw in the previous section, for a fixed dispersal rate the forward-in-time model produces a more clustered distribution of sample locations when there are more recombination nodes (Figure 5c). Consequently, for a given set of sample locations, incorporating more trees into the ARG (and therefore more recombination nodes) must necessarily lead to a corresponding increase in the estimated dispersal rate. Further, note that as long as we model movement along the edges of the loop by independent processes and condition on them meeting at the recombination node, we will observe similar behavior in dispersal estimates. Therefore, the problem of loops is not solely associated with Brownian motion.

To confirm that conditioning on lineages meeting at recombination nodes is the core cause of the bias in dispersal rate estimates, we next run simulations that are closer to Brownian motion. Our original simulations differ from Brownian motion in two main aspects: 1) they allow some distance between mates and 2) they have local density regulation. We address these separately. First, we decrease the average mating distance by reducing the variance of the mating kernel, $\sigma_{m}^{2}$ (Figure 5b). Second, we make density regulation more global by increasing the variance of the competition kernel, $\sigma_{c}^{2}$ (Figure 5c). Less localized competition leads to more clumping due to the “pain in the torus” (Felsenstein, 1975) and therefore indirectly decreases the average distance between mates. As a result, both of these modifications reduce the error in dispersal rate estimates and cause them to increase more slowly with the number of trees (Figure 5b–c).

We can also investigate the cause of the dispersal rate bias by modifying our model of inference. A key assumption is that we force parent lineages to meet exactly at recombination nodes. We completely relaxed this assumption by allowing parents to mate from any distance and placing the recombination node at their midpoint (Section S4.1). However, because the variance of the recombination node is reduced to a similar extent as in the original model (Figure 4b), we still see an increase in dispersal estimates as we increase the number of trees (Section S4.1). But, unlike in the original model, the variance along the parental lineages (e.g., node F in Figure 4b–iii), and consequently the variance along the lineages branching off from the loop (node C), are not reduced. This reduces the rate at which the dispersal estimate increases with the number of trees (Section S4.1).

### 4.3 Ancestor locations

To assess the accuracy of estimated ancestor locations (Equation 7), we estimate the location of random genetic ancestors within a simulated ARG of 1000 samples and compare to the truth. We select each genetic ancestor to locate by choosing a random sample, genome position, and time (e.g., the ancestor of sample 1 at genome position 1050bp, 200 generations in the past). We estimate a location using the ARG for the full chromosome (“ARG”), a local ARG containing 100 trees on either side of the tree at the chosen genome position (“Window”), and the local tree at the genome position (“Tree”). We also compare these estimates against those using the averaging-up approach (Wohns et al., 2022). For the averaging-up method, we first simplified our ARG before calculating the locations (Kelleher et al., 2018; Wong et al., 2023), as is done in practice (Wohns et al., 2022).

**Figure 6:**
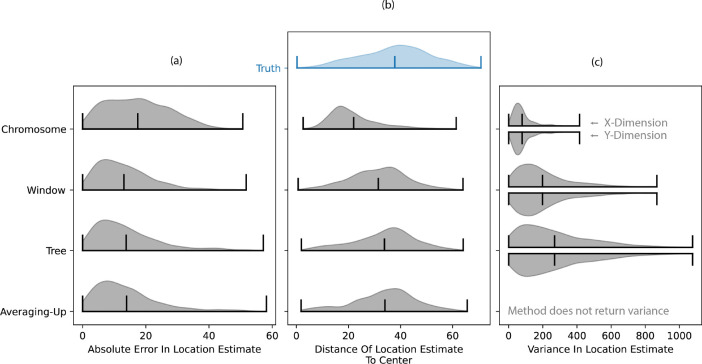
Accuracy of estimated ancestor locations. (a) Distribution of distances between the MLE ancestor locations and their true locations. Horizontal lines show the extremes and mean of each distribution. (b) Distributions of distances between the MLE ancestor locations and the average of the sample locations (”center”). The true distribution of distances of ancestor locations from the center is provided in blue for comparison. (c) Estimated variance in ancestor locations, computed using the effective dispersal rate from the simulation rather than the biased dispersal rate estimate.

Despite using more information, we observe larger absolute error with the ARG estimates than with the tree-based approach (Figure 6a). Investigating this further, we see that ancestors are often estimated too close to the average (“center”) of the sample locations (Figure 6b). Since the forward-in-time model sees excess clustering below recombination nodes (Figure 4b–ii, Figure 5d), backwards in time there is an equivalent pull towards the center (see Fig S10). This bias towards the center becomes more severe as more trees are incorporated into the ARG. Perhaps surprisingly, we see that the averaging-up method, which uses a simplified ARG and ignores edge lengths, performs just as well on average. A modified simulation with a much larger area and sampling from the center confirms this bias is not due to reflecting boundaries (Section S7).

Lastly, we see that the average uncertainty in location estimates gets smaller as we use more trees (Figure 6c). In practice the uncertainties would depend on the dispersal estimate but here we use the true dispersal rate to understand the unique properties of the location uncertainties, distinct from the issues of estimating dispersal. The uncertainty in location drops as we use more trees in part because each tree brings in more information. However, with more trees the model eventually becomes overconfident (Figure S9). Windowing may therefore be a promising direction forward. In these simulations at least, the number of trees in the window may be chosen so that the mean error is no worse than that from a single tree or the averaging-up method (Figure 6a) and the uncertainty is more accurate (Figure S9). However, there is currently no way of determining an appropriate number of trees to include in the window.

## 5 Promise of the approach

**Figure 7:**
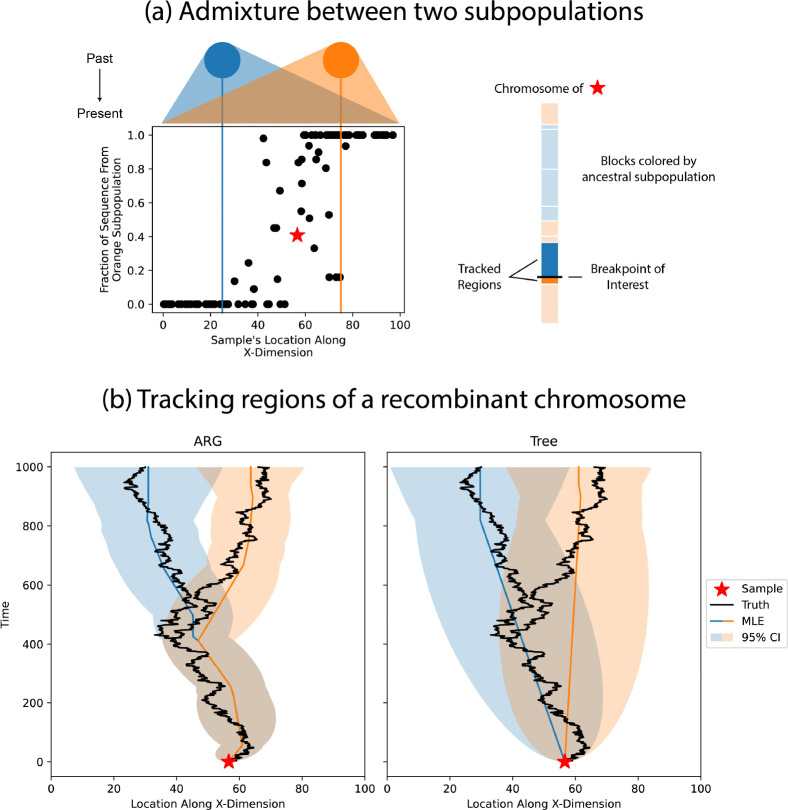
Visualizing the geographic history of admixture. We simulated two geographically isolated subpopulations dispersing into one another, leading to samples with genetic ancestors from both subpopulations. (a) At the top of subfigure is a cartoon of the dispersion of the subpopulations over time. The colored vertical lines mark the average starting position of each subpopulation along the X-dimension. As you look at samples positioned from left to right, the fraction of their sequence associated with the orange subpopulation increases. Regions of the starred sample’s chromosome are colored according to their associated ancestral subpopulation. The regions surrounding the breakpoint of interest are tracked in the following subfigure. (b) Two spatial reconstructions using the ARG for the full chromosome and the local trees immediately on either side of the breakpoint. The black lines mark the true locations of the lineages, the colored lines are the estimated most likely ancestral locations, and the shading is the 95% confidence interval around these estimates (using the effective dispersal rate from the simulation).

Acknowledging the problems illustrated above, we would like to end by illustrating the goal and promise of using an ARG-based approach for spatial reconstruction. Recombinant chromosomes show an intricate pattern of splits and merges with other sample lineages as they move through time and space. For example, in an admixed individual two regions of the chromosome will have identical spatial histories in the recent past, when they were inherited together, but very distinct spatial histories before they were brought together via recombination. To demonstrate this, we set up a simulation that starts with two geographically isolated subpopulations. Over 1000 generations, individuals in the two subpopulations disperse, intermingle, and mate in 2D. At the end of the simulation there is still a clear cline in genetic ancestry along the axis of separation between the original two subpopulations (Figure 7a). This cline is required for us to reconstruct the locations of genetic ancestors back to their original subpopulations. We then choose an arbitrary admixed individual and reconstruct the spatial histories of on either side of a recombination event. We highlight this breakpoint specifically because it separates genetic material from the two subpopulations, brought together 412 generations in the past. The ARG-based approach captures the “Y” pattern seen in the true locations, where following forward in time, the two lineages start in separate subpopulations before merging into a single path at the recombination event (Figure 7b). This pattern is not seen in estimates from individual trees as that approach does not take into account the sharing of edges between trees. Treating the two trees independently, the two lineages only come together at present day, where they meet at the sample. Other ARG-based approaches to spatial inference (Grundler et al., 2024; Wohns et al., 2022) can also capture the splitting of lineages at recombination events but do not provide measures of uncertainty, which hinders the development of future hypothesis tests.

## 6 Data Availability

Our method is available as a Python package at https://github.com/osmond-lab/sparg. The code for all of our analyses in this paper is available in the “manuscript” branch of the GitHub repository (https://github.com/osmond-lab/sparg/tree/manuscript).

## 7 Discussion

We generalized a model of Brownian motion on trees to estimate dispersal rates and locate genetic ancestors from an ARG, and developed algorithms to do this efficiently. The full ARG provides more information than previous tree-based methods (Figure 2) and therefore estimates the location of genetic ancestors with more certainty (given the same dispersal rate; Figure 3). The great promise of such an approach is to geographically track a sample’s genetic ancestry back in time as it splits and merges via recombination and coalescence, with uncertainties. This could be particularly exciting for visualizing the divergent geography history of admixed samples (Figure 7) or locating recombination nodes of interest, e.g., viral recombination events implicated in epidemics (Ignatieva et al., 2022; Tamura et al., 2023).

Despite the promise, we discovered an interesting but unfortunate property of the model: the dispersal estimate increases monotonically as we use more marginal trees (Figure 5a). We narrowed down the root cause of this, both through simulations and mathematical proofs, to a reduction in the variance of loop node locations (and consequently that of descendant samples) under a Brownian motion model (Figure 4, Figure 5c, Section S5). Our model assumes that two lineages precisely meet at a recombination node, which is an unlikely event under Brownian motion (Etheridge, 2019), especially if they were ever far apart. This implies that the two lineages must not have dispersed very far from each other since their most recent common ancestor, pulling all nodes in and below a recombination loop closer together (Figure 5c). Consequently, given the same sample locations, each additional recombination loop increases our dispersal estimate. As each additional tree comes with an additional recombination loop, our dispersal estimate increases with the number of trees (Figure 5a). Ancestral location estimates were also shown to have a bias towards the center due to this behavior (Figure 6). Allowing the parents of a recombination node to be any distance apart reduced the rate that dispersal estimates increased with the number of trees (Section S4.1) but did not solve the problem because placing the recombination node at the average of the two parents reduced its variance.

The problem of loops poses a key challenge in utilizing ARGs for spatial inference. Despite the “pain in torus” (Felsenstein, 1975), simple models of Brownian motion often provide accurate spatial inference on trees (Bradburd and Ralph, 2019; Novembre and Slatkin, 2009). However, here we have shown that the additional and distinct problem of loops makes these simple Brownian motion models less accurate for spatial inference when extended to work on ARGs. We are therefore still without an analytically tractable (or at least computationally efficient) model that can utilize the complete information encoded in an ARG and provide accurate ancestor location estimates with confidence intervals. From the insights gained from this project, we highlight some ways forward given this problem of loops.

One way to have less biased parameter estimates is to use a more accurate model. An obvious choice is the spatial Λ-Fleming-Viot (SLFV) process (Barton et al., 2010a,b). By modeling local density dependence (which allows two lineages of a loop to be non-independent even before conditioning them on meeting) and non-zero mating distance (relaxing the meeting condition) this approach gives more accurate estimates than simple Brownian motion when applied to trees (Kalkauskas et al., 2021). The SLFV model can be extended to incorporate recombination between lineages that are not at the exact same location (Barton et al., 2010b; Etheridge and Véber, 2012). One of the key steps in utilizing the SLFV model for inference with ARGs is to extend the model to incorporate recombination along a continuous genome (as opposed to the two loci; Etheridge and Véber (2012)) which, while promising, is a difficult problem. Further, the complexity of this model makes it computationally intensive for inference and therefore limited to small sample sizes, even in the case of no recombination (Guindon et al., 2016). It may then be helpful to think of ways to develop more tractable and scalable models. In doing so, it is worth noting that the problem of loops is not specific to Brownian motion. In fact, as long as movement along edges of a loop are modeled as independent processes with restrictions coming solely due to what happens at recombination nodes, we will see an increased clustering of tip locations with the number of recombination nodes. One possible way around this is to model movement with Levy processes that are not independent to begin with, for instance compound Poisson processes, which show up as limiting cases of the SLFV (Barton et al., 2010a; Berestycki et al., 2009). Another option would be to use machine learning algorithms to correct for model misspecification. This may be especially doable here where the dispersal rate estimate increases nearly linearly with the number of marginal trees (Figure 5a), so that correction only requires knowing the slope.

Brownian models of spatial movement on coalescent trees are identical to Brownian models of trait evolution on phylogenetic trees Bradburd and Ralph (2019); Neigel and Avise (1993). Phylogenetic networks, where reticulations are due to hybridization, gene flow, or introgression, potentially provide a similar structural counterpart to ARGs. Unlike in the case of trees though, there is an important difference worth keeping in mind. With spatial movement on an ARG, two parental lineages are expected to be near one another immediately above a recombination node but when modeling trait evolution on a phylogenetic network the mean trait values of two parental populations can be arbitrarily far apart when the two populations hybridize. This is the key difference between our model and that of Bastide et al. (2018), which models trait evolution on phylogenetic networks as we do in our alternate model (Section S4.1), where the parents of a recombination node can be any distance apart. Although this difference (parents being physically close, hybridizing populations being arbitrarily far in trait space) is the norm, there are exceptions in both situations (e.g., long-range pollen dispersal allows parents to be far apart and genetic barriers to gene flow can restrict how different parental populations can be in trait space), which strengthens the case for more crosstalk between population genetics and phylogenetics. For example, the problem of loops is not a problem for modeling trait evolution on phylogenetic networks given the correct phylogenetic network because the model is not (necessarily) misspecified. However, our results imply that the estimated rate of evolution is extremely sensitive to the number of reticulations in the network, which is known to be difficult to estimate accurately.

In conclusion, we have developed a mathematically rigorous and tractable model that uses the complete genealogical history of a set of samples to reconstruct the spatial history of their genetic ancestors, with uncertainties. While such an approach holds great promise, for example to visualize the geographic history of admixed samples, we see that our estimates are biased. We demonstrate that the problem is reduced variation at and below recombination loops under the model. This makes the ubiquitous model of Brownian motion inaccurate for spatial inference on ARGs, leaving a gap for future tractable and computationally-efficient methods to fill.

## Supplementary Material

Supplement 1

## Figures and Tables

**Table 1:** Description of key symbols used.

Symbol	Name	Description (and notes)
$n_{s}$	Number of samples	
$n_{p}$	Number of paths	Total number of paths in the ARG from a sample to a root
$n_{r}$	Number of roots	
$\sigma^{2}$	Dispersal rate	Variance in offspring locations around parent
$S_{p}$	Path matrix	Shared times between each pair of paths (could refer to either the full path matrix or the minimal path matrix)
S	Sample matrix	$\sigma^{2}S$ is covariance matrix between sample locations
P	Path-sample matrix	$n_{p}\times n_{s}$ matrix whose entry is 1 if path i is associated with sample j
R	Path-root matrix	$n_{p}\times n_{r}$ matrix whose entry is 1 if path i is associated with root j
$\overset{\to }{\ell^{*}}$	Observed sample locations	Vector of length $n_{s}$
$\vec{L}$	Sample location vector	Vector-valued random variable of sample locations
$\vec{L_{p}}$	Path location vector	Vector-valued random variable of sample locations at the end of each path
$\overset{\to }{\mu }$	Root location vector	Vector of length $n_{r}$
$L_{a}$	Internal node location	Random variable denoting location of internal node a
$\overset{\to }{s_{a}}$		Vector-valued random variable with shared time between the path of internal node s and each path in $S_{p}$
V	Ancestor specific uncertainty	Component of location variance related to the position of the ancestor in the ARG and root uncertainty (not dispersal)
$t_{a}$		Time from node a to the root
